# Supplementary material for: Optimized Selective Media Enhance the Isolation and Characterization of Gut-Derived Probiotic Yeasts
Source: J Fungi (Basel). 2025 Dec 15;11(12):885. doi: 10.3390/jof11120885 (PMC12733629; doi:10.3390/jof11120885)
Supplement: Supplementary file 1 [file jof-11-00885-s001.zip › jof-3984711-supplementary.pdf]

Table S1. Yeast recovery rates for all medium combinations.

	Log CFU/ mL											
	MSA-Dose A			MSA-Dose B			Dixon-Dose A			Dixon-Dose B		
Temperature (°C)	D1	D2	D3	D1	D2	D3	D1	D2	D3	D1	D2	D3
30	2.9934	2.9294	2.9294	4.1959	4.2014	4.1987	4.0212	3.9800	3.7889	4.3608	4.3883	4.4273
37	3.1446	3.0920	3.1446	5.2148	5.2504	5.2253	4.4346	4.4518	4.2833	5.3802	5.2672	5.2418

Table S2. List of safety assessment results of yeast isolates.

#	Blood agar	DNAse	Coagulase	Isolate Code
1	Positive	Negative	Positive	Y186
2	Positive	Positive	Positive	Y77
3	Positive	Positive	Positive	Y78
4	Positive	Positive	Positive	Y80
5	Positive	Negative	Positive	Y187
6	Positive	Negative	Positive	Y188
7	Positive	Negative	Positive	Y189
8	Positive	Negative	Positive	Y190
9	Positive	Positive	Positive	Y86
10	Positive	Positive	Positive	Y87
11	Positive	Positive	Positive	Y89
12	Positive	Negative	Positive	Y191
13	Negative	Positive	Positive	Y167
14	Negative	Positive	Positive	Y168
15	Positive	Negative	Positive	Y165
16	Positive	Positive	Positive	Y90
17	Positive	Positive	Positive	Y113
18	Positive	Positive	Positive	Y117
19	Positive	Negative	Positive	Y193
20	Positive	Negative	Positive	Y194
21	Positive	Negative	Positive	Y195
22	Positive	Negative	Positive	Y196
23	Positive	Positive	Positive	Y129
24	Positive	Positive	Positive	Y200
25	Positive	Negative	Positive	Y197
26	Positive	Negative	Positive	Y166
27	Negative	Positive	Positive	Y169
28	Negative	Positive	Positive	Y170

29	Negative	Negative	Positive	Y171
30	Positive	Positive	Positive	Y181
31	Positive	Positive	Positive	Y182
32	Positive	Positive	Positive	Y183
33	Positive	Positive	Positive	Y184
34	Positive	Positive	Positive	Y185
35	Negative	Negative	Positive	Y172
36	Negative	Positive	Positive	Y173
37	Negative	Negative	Positive	Y174
38	Negative	Negative	Negative	Y6
39	Negative	Negative	Negative	Y22
40	Negative	Negative	Negative	Y42
41	Negative	Negative	Negative	Y48
42	Negative	Negative	Negative	Y55
43	Negative	Negative	Negative	Y56
44	Negative	Negative	Negative	Y73
45	Negative	Negative	Negative	Y105
46	Negative	Negative	Negative	Y127
47	Negative	Negative	Negative	Y44
48	Negative	Negative	Negative	Y59
49	Negative	Negative	Negative	Y7
50	Negative	Negative	Negative	Y8
51	Negative	Negative	Negative	Y34
52	Negative	Negative	Negative	Y69
53	Negative	Negative	Negative	Y74
54	Negative	Negative	Negative	Y101
55	Negative	Negative	Negative	Y109
56	Negative	Negative	Negative	Y111
57	Negative	Negative	Negative	Y115
58	Negative	Negative	Negative	Y130
59	Negative	Negative	Negative	Y192
60	Negative	Negative	Negative	Y200
61	Negative	Negative	Negative	Y201
62	Negative	Negative	Negative	Y5
63	Negative	Negative	Negative	Y10
64	Negative	Positive	Negative	Y66
65	Negative	Positive	Negative	Y79
66	Negative	Negative	Positive	Y67
67	Negative	Negative	Positive	Y68
68	Negative	Negative	Positive	Y83
69	Negative	Negative	Positive	Y99
70	Negative	Negative	Positive	Y101
71	Negative	Negative	Positive	Y103
72	Negative	Negative	Positive	Y108

73	Negative	Negative	Positive	Y110
74	Negative	Negative	Positive	Y119
75	Negative	Negative	Positive	Y2
76	Negative	Negative	Positive	Y4
77	Negative	Negative	Positive	Y11
78	Negative	Negative	Positive	Y18
79	Negative	Negative	Positive	Y19
80	Negative	Negative	Positive	Y20
81	Negative	Negative	Positive	Y21
82	Negative	Negative	Positive	Y30
83	Negative	Negative	Positive	Y71
84	Negative	Negative	Positive	Y72
85	Negative	Negative	Positive	Y75
86	Negative	Negative	Positive	Y35
87	Negative	Negative	Positive	Y36
88	Negative	Negative	Positive	Y37
89	Negative	Negative	Positive	Y43
90	Negative	Negative	Positive	Y44
91	Negative	Negative	Positive	Y45
92	Negative	Negative	Positive	Y46
93	Negative	Negative	Positive	Y47
94	Negative	Negative	Positive	Y59
95	Negative	Negative	Positive	Y76
